# The predictive effect of platelet recovery on the prognosis of severe fever with thrombocytopenia syndrome

**DOI:** 10.3389/fcimb.2025.1644207

**Published:** 2025-11-04

**Authors:** Bing Tian, Yuan Gao, Qiyue Sheng, Xuelan Mao, Jinyong Wang, Shujun Zhang, Jingxia Peng, Lijia Li, Yumeng Hou, Jingyi Chen, Zhiqian Wang, Yu Di, Bo Zhou, Baocheng Deng

**Affiliations:** ^1^ The Second Department of Infectious Diseases, The First Affiliated Hospital, China Medical University, Shenyang, Liaoning, China; ^2^ Department of Infectious Disease, Zhongda Hospital Southeast University, Nanjing, Jiangsu, China; ^3^ National Clinical Research Center for Laboratory Medicine, Department of Laboratory Medicine, The First Hospital of China Medical University, Shenyang, Liaoning, China; ^4^ Department of Infectious Disease, Jinhua Municipal Central Hospital, Jinhua, Zhejiang, China; ^5^ Department of Gastroenterology, Guiqian International General Hospital, Guiyang, Guizhou, China; ^6^ Department of Infectious Disease, Kuandian Country Hospital, Dandong, Liaoning, China; ^7^ Department of Infectious Disease, Fengcheng Center Hospital, Dandong, Liaoning, China; ^8^ Clinical College of Ophthalmology, School of Medicine, Nankai University, Tianjin, China; ^9^ School of Public Health, China Medical University, Shenyang, Liaoning, China; ^10^ Department of Ophthalmology, Shengjing Hospital of China Medical University, Shenyang, Liaoning, China; ^11^ Department of Clinical Epidemiology and Evidence-Based Medicine, The First Hospital of China Medical University, Shenyang, Liaoning, China

**Keywords:** severe fever with thrombocytopenia syndrome, platelet recovery, prognosis, case fatality rate, nomogram

## Abstract

**Background:**

Severe fever with thrombocytopenia syndrome (SFTS), an emerging infectious disease, has a high case fatality rate (CFR) in severe patients. Thrombocytopenia is one of the features of SFTS, and a platelet count lower than 50×10^9^/L is a risk factor for mortality in patients with SFTS. However, there have been no studies on the value of platelet recovery in the prognosis of SFTS patients.

**Methods:**

From January 2009 to December 2020, laboratory-confirmed severe SFTS patients with platelet counts lower than 50×10^9^/L were enrolled and divided into a survival group and a death group based on 90-day prognosis. Descriptive analysis of baseline data compared characteristics between the survival and death groups. Multivariate Cox proportional hazards regression models identified independent mortality risk factors for SFTS patients. A nomogram-presented prediction model was constructed via multivariate Cox regression. The performance of nomogram was measured by the receiver operating characteristic (ROC) curve, calibration diagram, and decision curve analysis (DCA).

**Results:**

144 SFTS patients with platelet counts< 50×10^9^/L during the disease were included. After three months of follow-up, 109 patients survived and 35 patients died. The cut-off values for predicting fatal outcomes were 40×10^9^/L for platelet levels on day three (PLT Day3) and 50×10^9^/L for platelet levels on day five (PLT Day5), respectively. Statistical analysis showed a significant difference (p<0.001) in platelet recovery to these levels within 3 or 5 days. Kaplan-Meier analysis showed that patients with unrecovered PLT on day 5 had a higher cumulative incidence of mortality than those with recovered PLT on day 5. Multivariate Cox regression found age ≥65, failure of platelet count to reach 50×10^9^/L in 5 days, prolonged APTT, and elevated LDH as independent mortality risk factors (p<0.05). Subgroup analysis showed a significant association between whether the platelet count recovers to 50×10^9^/L within 5 days (PLT-Day5) and mortality in all subgroups.

**Conclusions:**

Whether the platelet count recovers to 50×10^9^/L within 5 days (PLT-Day5), aspartate aminotransferase (APTT), lactate dehydrogenase (LDH) and age are independent predictors of mortality in severe SFTS patients with platelet counts below 50×10^9^/L during the course of the disease. Patients whose platelet count recovers from the lowest value to 50×10^9^/L within five days have a better prognosis.

## Background

Severe fever with thrombocytopenia syndrome virus (SFTSV), commonly referred to by its scientific name Bandavirus dabieense, is a tick-transmitted virus. It is classified under the genus Bandavirus, which falls within the family Phenuiviridae and the order Bunyavirales (Casel et al., 2021). Infection with SFTSV can lead to severe fever with thrombocytopenia syndrome (SFTS), whose clinical features include acute high fever, thrombocytopenia, leukopenia, increased serum liver enzymes, gastrointestinal symptoms, and multiple organ failure (Yu et al., 2011).

Since its first identification, SFTSV has shown a gradually expanding geographic distribution. SFTS was first reported in China in 2009 (Fang et al., 2015), followed by South Korea in 2010 (Kim et al., 2013) and in Japan in 2013 (Takahashi et al., 2014). Since 2019, SFTSV infection has also been first reported in Southeast Asia (in Vietnam in 2019 (Tran et al., 2019), in Myanmar (Win et al., 2020), Thailand (Daengnoi et al., 2020) and Pakistan (Zohaib et al., 2020) in 2020). Domestically in China, SFTS is primarily endemic in hilly areas (Zhan et al., 2017); as of now, SFTSV infection cases have been reported in 27 provinces (Peng et al., 2025). Epidemiological data further highlight its public health threat: seroprevalence studies show that approximately 4.3% of residents in Chinese SFTS-endemic areas have been infected with SFTSV, and annual reported SFTS cases in China ranged from 1,000 to 2,500 between 2011 and 2016 (Reece et al., 2018).

Clinically, SFTS presents significant challenges: even among patients with similar initial symptoms who receive identical treatment, outcomes vary substantially, with overall case fatality rates (CFRs) ranging from 6.4% to 20.9% (Kim et al., 2019). Notably, our previous research found that the CFR of severe SFTS patients reaches approximately 34% (Deng et al., 2013). Currently, the pathogenesis of SFTSV remains poorly understood, and no effective antiviral agents are available—making early identification of risk factors for critical illness a critical priority.

Thrombocytopenia is a key hallmark of SFTS. Prior studies have identified a platelet count below 50×10^9^/L as a risk factor for poor prognosis in SFTS patients (Cui et al., 2012). However, the role of platelet recovery (a dynamic indicator of disease progression) in predicting SFTS prognosis has not been investigated. To address this research gap, we conducted a retrospective analysis to explore the relationship between platelet recovery and prognosis in patients with severe SFTS.

## Methods

### Ethical statement

This study was approved by the medical science research ethics committee of The First Affiliated Hospital of China Medical University. The First Hospital of China Medical University served as the lead institution for this study. The participating institutions included Kuandian County Hospital, Fengcheng Center Hospital, and Jinhua Municipal Central Hospital. Prior to the collection of clinical data and specimens, informed consent was obtained from all patients. Additionally, each participating institution received approval from their respective ethics committees.

### Study population and data collection

From January 2009 to December 2020, laboratory-confirmed SFTS patients from four hospitals in China (The First Hospital of China Medical University, Kuandian County Hospital, Fengcheng Center Hospital, and Jinhua Municipal Central Hospital) were collected for a retrospective cohort study. Demographic parameters include: admission date, discharge or death date, age, and gender. Laboratory tests include: routine biochemical, hematological, and coagulation results. For biochemical indicators other than platelets, the detection time points are all at admission. According to the 90-day prognosis, patients were divided into a survival group and a death group. Trained input personnel compiled the information in Excel spreadsheets and established a case information database.

### Inclusion criteria

1. Patients met the criteria for severe SFTS diagnosis; 2. Patients with a platelet count of less than 50×10^9^/L.

### Exclusion criteria

1. Laboratory-confirmed infections by other pathogens, such as Platts and Mori rickettsia, Orientia tsutsugamushi, and Hantavirus; 2. Human granulocytic anaplasmosis; 3. A history of acute or chronic blood system diseases; 4. Autoimmune diseases; 5. Patients who received therapeutic platelet transfusion within 5 days after their platelet count first dropped below 50×10^9^/L.

### Laboratory diagnostic criteria meet any of the following conditions

1. Viral nucleic acid is detectable by real-time RT-PCR; 2. Immunoglobulin M (IgM) antibody in acute-phase serum is positive for SFTS.

### SFTSV RNA detection

RT-FQ-PCR was conducted with the Fluorescence Polymerase Chain Reaction (PCR) Diagnostic Kit for Severe Fever with Thrombocytopenic Syndrome Bunyavirus (SFTSV) RNA (Zhongshan Bio-Tech Co., LTD, Guangdong, China). ABI Prism 7500 was used for amplification. Reaction parameters were 1 cycle of 50 °C for 15 min, 1cycle of 95 °C for 15 min, and then 45 cycles of 94 °C for 15 s and 55 °C for 45 s. The cut-off cycle threshold (Ct) value was set at 35 cycles.

### SFTSV antibody detection

Serum SFTSV-specific IgM antibody was detected using an ELISA kit (Zhongshan Bio-Tech Co., LTD, Guangdong, China) according to the manufacturer’s instructions.

### Severe case definition

Laboratory-confirmed cases admitted to the intensive care unit during the course of the disease and one of the following cases is a severe case (Deng et al., 2012).

1. Acute lung injury or acute respiratory distress syndrome
2. Acute heart failure
3. Encephalitis
4. Shock
5. Septicemia
6. Disseminated intravascular coagulation (DIC)
7. Death

### Platelet count testing

Platelet count was tested daily when it decreased to 50×10^9^/L. Platelet count was tested every two or three days when the platelet count was higher than 50×10^9^/L and less than 100×10^9^/L.

### Statistical analysis

**Figure 1:**
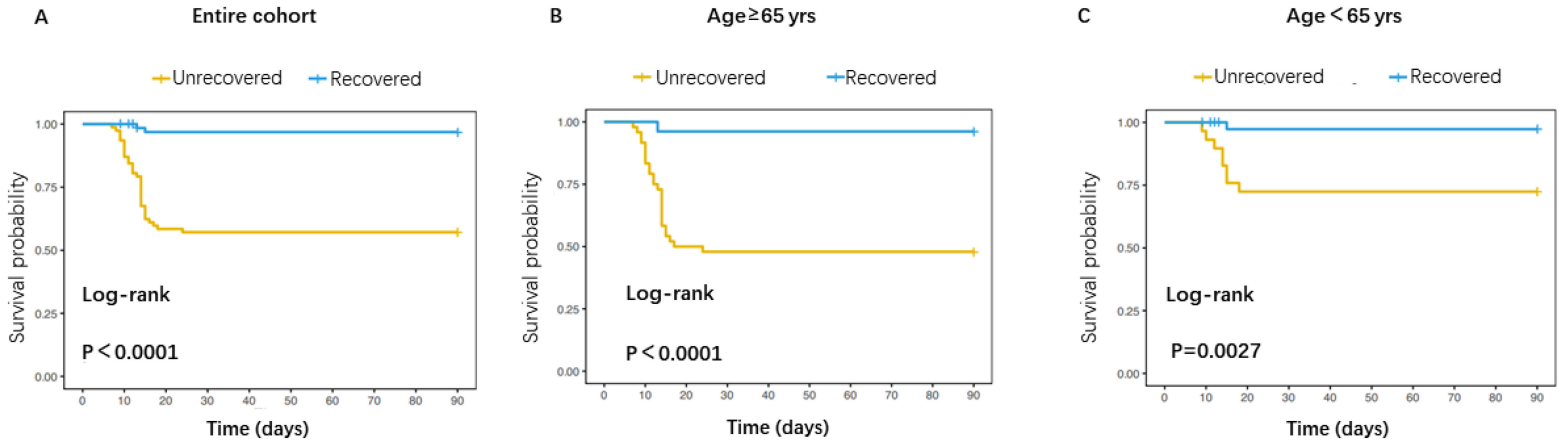
Comparison of mortality risk between patients with recovered and unrecovered PLT on day 5. **(A)** Survival curve for the overall patients; **(B)** Survival curve for patients aged≥65 years; **(C)** Survival curve for patients aged<65 years.

**Figure 2:**
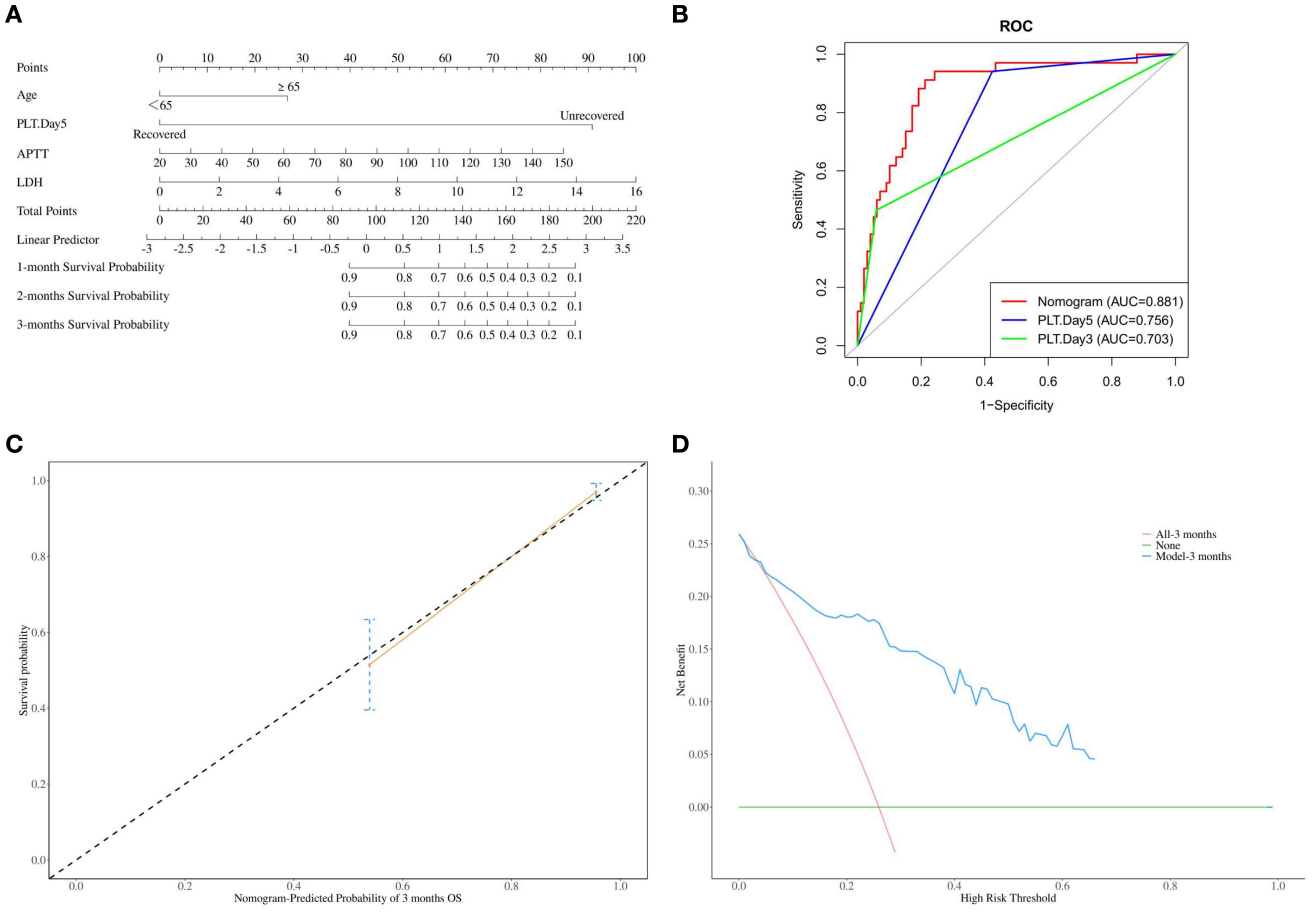
**(A)** Nomogram for predicting the 1-month, 2-month, and 3-month mortality rates of severe SFTS patients; **(B)** The area under the curve (AUC) for nomogram, PLT-Day3 and PLT-Day5 prediction of mortality in severe SFTS; **(C)** Calibration curve of nomogram for the 3-month mortality rates of severe SFTS patients; **(D)** The decision curve analysis (DCA) for the model.

**Figure 3:**
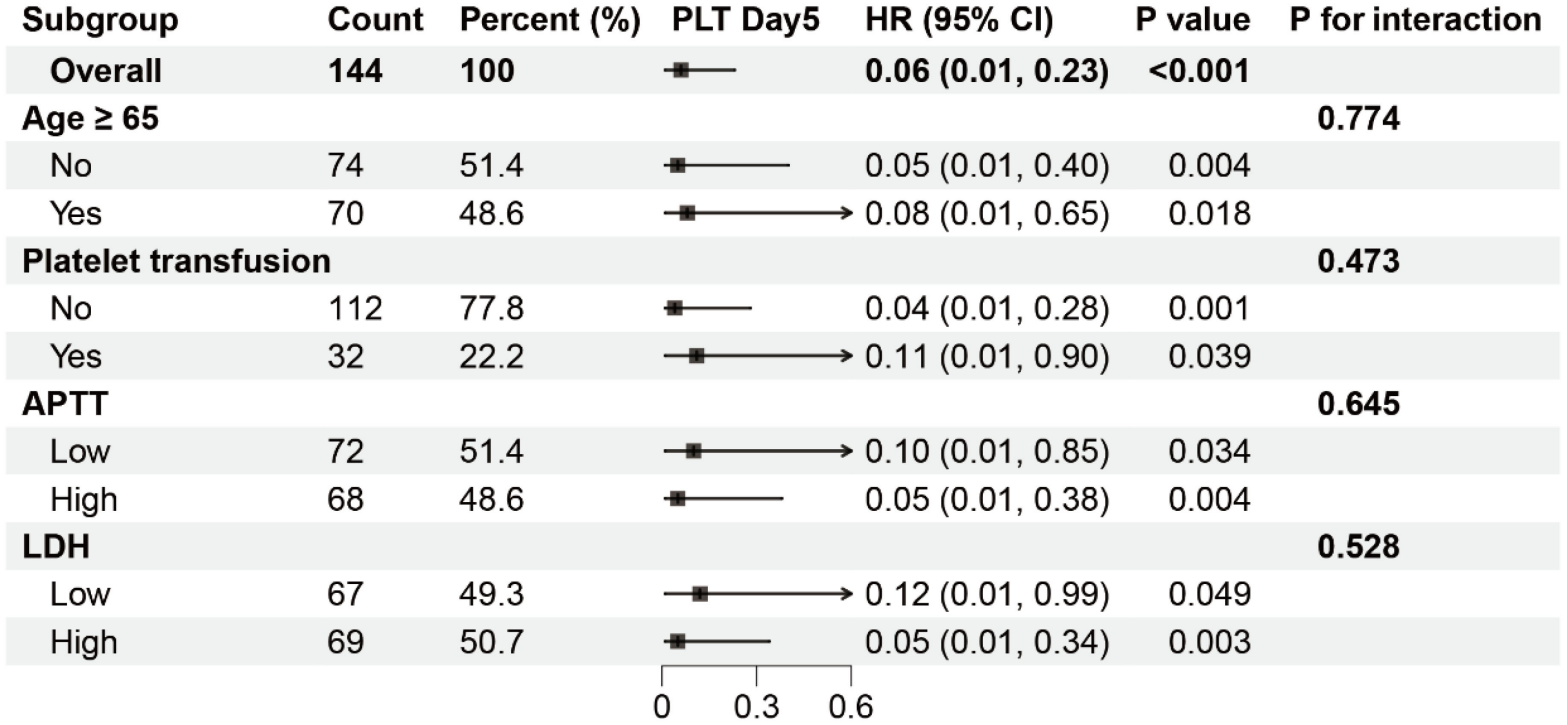
Subgroup analysis of the association between PLT-Day5 and mortality in severe SFTS patients. HR, hazard ratio; CI, confidence interval. The patients were divided into two groups, Low and High, based on the median.

The results are expressed as median (interquartile range, IQR) and percentage. We compared the data of the death and survival groups in severe SFTS cases. The independent sample *t*-test was used to compare the mean values of continuous variables, where the data were normally distributed. Otherwise, we used the Mann-Whitney test. The proportion of the categorical variables was compared using the chi-square test. Receiver operating characteristic (ROC) curve analysis was used to determine the optimal cut-off values of platelet counts on Day 3 and Day 5 for predicting mortality outcomes. The cut-off values were selected based on maximizing the Youden index. For survival analysis, a multivariate Cox proportional hazards regression model was employed to evaluate the independent effects of various factors on mortality risk. This model was chosen because it can appropriately handle censoring, a common issue in survival time data, and allows for the simultaneous analysis of the impact of multiple predictive variables on the hazard rate. Meanwhile, covariates were evaluated based on residual test results, confirming that the proportional hazards assumption was satisfied. The Kaplan-Meier method and the Log-rank test were adopted to compare survival differences among patients grouped by whether their platelet count recovers to 50×10^9^/L within 5 days. Based on univariate analysis, collinearity test results, and clinical experience, variables potentially associated with mortality in severe SFTS patients were selected and included in the multivariate Cox regression analysis model. The pairwise comparison of the model’s ROC curves was performed using DeLong’s test. Statistical analyses were performed using SPSS, version 27.0, software (IBM, Armonk, NY, USA). **Figures 1**–**3** were plot by RStudio version 4.2.1.

## Result

### Demographic and clinical characteristics of severe SFTS patients

**Figure 4:**
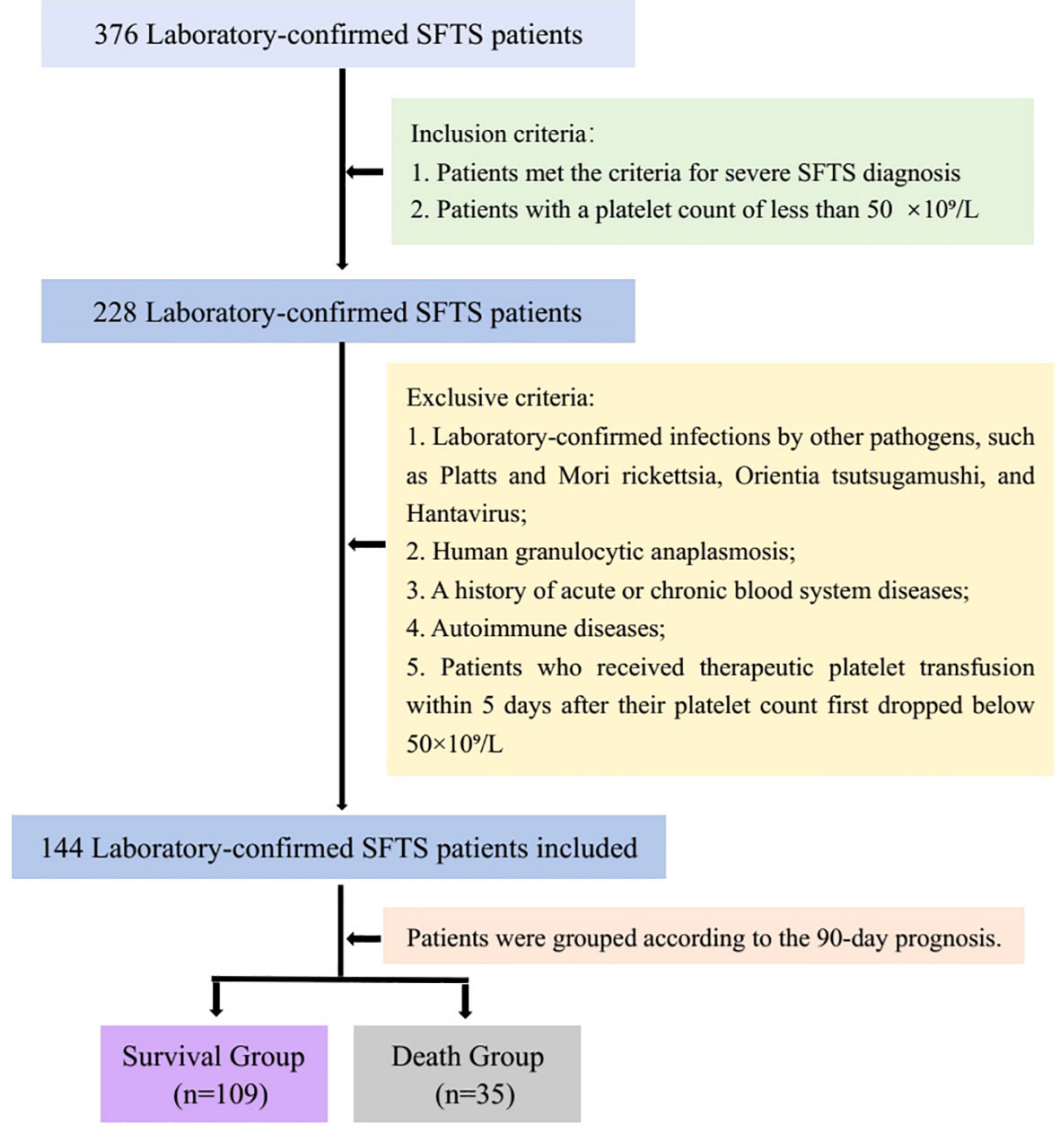
The flow chart of patient inclusion and exclusion was shown in **Figure 4**.

A total of 376 cases were diagnosed as having SFTS by RT-PCR and/or ELISA. Finally, 144 cases were included in the study and were divided into survival group (109 cases) and death group (35 cases) (**Figure 4**). Most of the patients were farmers and lived in rural and hilly areas. Thirty-five (24.3%) patients died. The survival group consisted of 45 (41.3%) women and 64 (58.7%) men, while the death group comprised 13 (37.1%) women and 22 (62.9%) men. There was no difference in gender composition between the two groups. In the death group, the median age was 69.0 years (IQR, 63.5-76.5). In the survival group, the median age was 63.0 years (IQR, 56.0-70.0). The proportion of patients older than 65 years was significantly higher in the death group compared to the survival group (*p* < 0.001). The main clinical features of severe patients with SFTS were fever (n = 144, 100%) and thrombocytopenia (n = 144, 100%). Patients with underling diseases accounted for 17.1% (6/35) of the death group and 18.3% (20/109) of the survival group, with no significant difference between the two groups (*p* = 0.897). There was no difference between the survival and death groups in terms of whether they received platelet transfusions (**Table 1**).

**Table 1:** Comparison of general conditions between survival group and death group.

Parameters	Total	Survival	Death	P Value
No. of patients	144	109	35	–
Gender, No. (%)		–	–	–
Male	86 (59.7)	64 (58.7)	22 (62.9)	0.173
Female	58 (40.3)	45 (41.3)	13 (37.1)	
Age, median (IQR), year	65 (57.0-72.0)	63.0 (56.0-70.0)	69.0 (63.5-76.5)	<0.001
Age≥65 years, No. (%)	75 (52.1)	49 (45.0)	26 (74.3)	<0.001
Age<65 years, No. (%)	69 (47.9)	60 (55.0)	9 (25.7)	
Underling disease ^a^, No. (%)	26 (18.1)	20 (18.3)	6 (17.1)	0.897
Platelet transfusions ^b^, No. (%)				
Yes	32 (22.2)	22 (20.2)	10 (28.6)	0.299
No	112 (77.8)	87 (79.8)	25 (71.4)	

a Underling diseases include hypertension, cerebrovascular disease, diabetes, chronic hepatitis, arrhythmia, and chronic bronchitis.

b All platelet transfusions reported in **Table 1** occurred more than 5 days after the platelet count dropped below 50×10^9^/L.

IQR, interquartile range.

**Figure 5:**
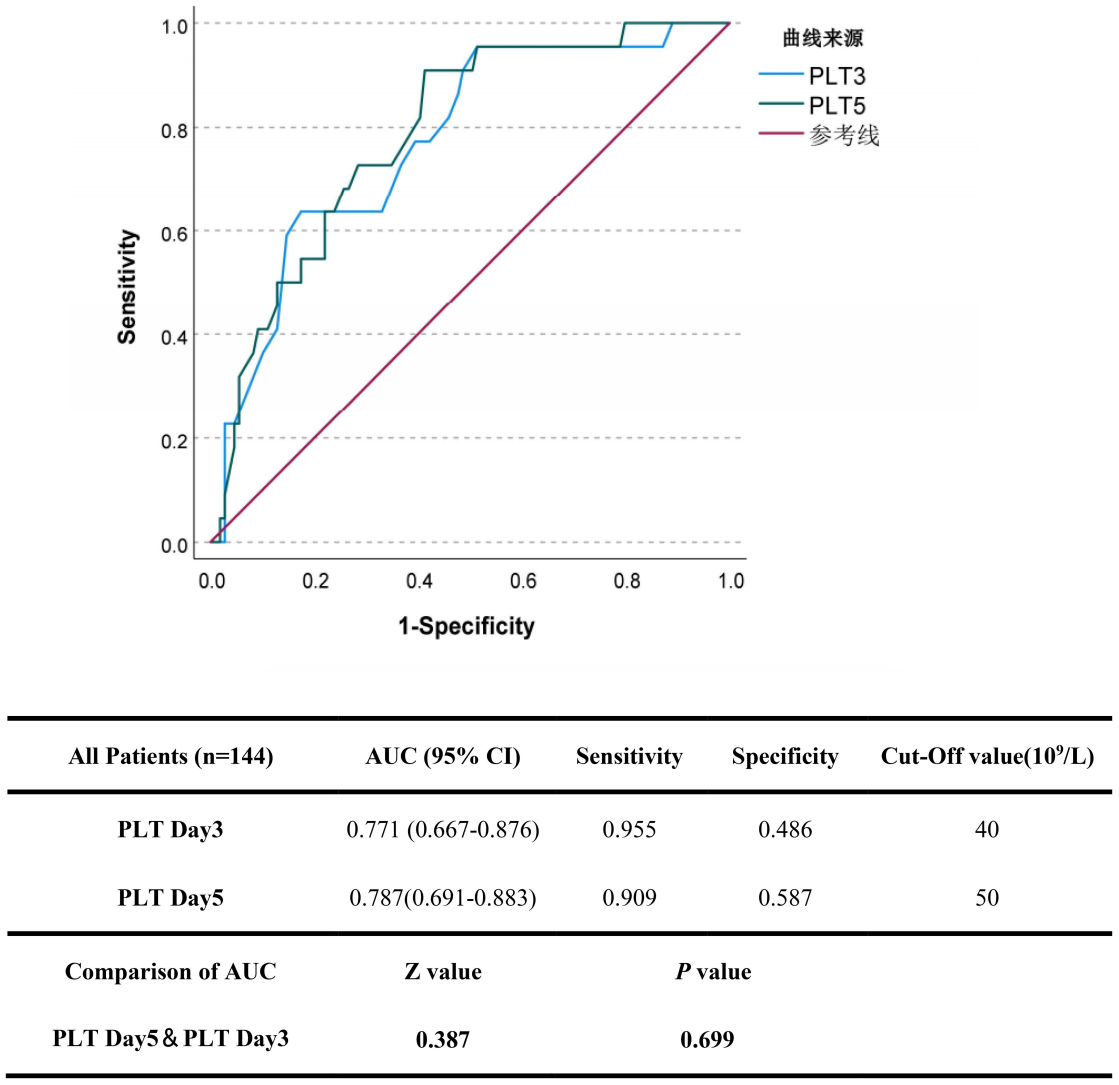
The predictive accuracy of platelet recovery indicators in severe SFTS patients. The efficacy of platelet recovery indicators (PLT Day3 and 5) in predicting the fatal outcome of patients with SFTS was calculated using receiver operating characteristic (ROC) curves.

To evaluate the effectiveness of platelet count recovery in predicting fatal outcomes in patients with SFTS, platelet counts on Day 3, and Day 5 were included in the ROC analysis. As shown in **Figure 5**, the cut-off values for predicting fatal outcomes were 40×10^9^/L for PLT Day3 and 50×10^9^/L for PLT Day5, respectively. The AUC of the PLT Day5 was the highest (0.787), followed by the PLT Day3 (0.771). The difference in AUC between PLT Day 5 and PLT Day 3 was not significant.

### Biochemical and hematological test results of survival and death groups of severe patients with SFTS

In the death group, the median white blood cell count was 1.67×10^9^/L (IQR, 1.23-2.55×10^9^/L), while the proportion of patients whose platelets recovered to 40×10^9^/L within three days after platelets decreased to 50×10^9^/L was 5.7% (2/35), and the proportion of patients whose platelets recovered to 50×10^9^/L within five days was 5.7% (2/35). In the death group, no patients died before the 3-day evaluation period, but 13 patients died between days 3 and 5. Therefore, when assessing platelet recovery on day 5, these 13 patients are considered to have had no platelet recovery. In the survival group, the median white blood cell level count was 2.10×10^9^/L (IQR, 1.39-3.05×10^9^/L), while the proportion of patients whose platelets recovered to 40×10^9^/L within three days after platelets decreased to 50×10^9^/L was 47.7% (52/109), and the proportion of patients whose platelets recovered to 50×10^9^/L within five days was 59.6% (65/109). Statistical analysis showed a significant difference (*p* < 0.001) between the two groups in whether the platelet count recovered to 40×10^9^/L within three days or to 50×10^9^/L within five days. However, the white blood cell count was not statistically significant between the two groups (*p* = 0.253). Some patients demonstrated liver injury with elevated aspartate aminotransferase (AST) and alanine aminotransferase (ALT). In the death group, the median AST was 304.0 U/L (IQR, 85.9-554.3 U/L) and the median ALT was 93.8 U/L (IQR, 44.5-148.0 U/L). In the survival group, the median AST was 178.7 U/L (IQR, 97.5-338.5 U/L) and the median ALT was 89.8 U/L (IQR, 52.0-162.9 U/L). There was no significant difference in viral load between the death group (4.5 Log_10_ copies/mL [IQR, 4.3-4.7 Log_10_ copies/mL]) and the survival group (4.6 Log_10_ copies/mL [IQR, 4.4-4.8 Log_10_ copies/mL]) (*p* = 0.178). The levels of creatine kinase (CK) and lactate dehydrogenase (LDH) were significantly increased, and aspartate aminotransferase (APTT) was markedly prolonged in the death group compared with the survival group. Whether the platelet count recovers to 40×10^9^/L within 3 days (PLT-Day3) and whether the platelet count recovers to 50×10^9^/L within 5 days (PLT-Day5) showed significant differences between the survival group and the death group (*p* < 0.001) (**Table 2**).

**Table 2:** Comparison of blood biochemical tests during hospitalization between survival group and death group on admission.

Parameters^*†a^	Survival	Death	P Value
APTT, median (IQR), s	44.8 (38.7-53.0)	59.9 (51.4-69.4)	<0.001
PT, median (IQR), s	12.6 (11.4-13.4)	12.9 (11.9-14.2)	0.321
CK, median (IQR), U/L	438.6 (153.0-931.9)	708.0 (289.0-1291.0)	0.041
LDH, median (IQR), U/mL	0.9 (0.2-15.6)	1.8 (1.1-3.2)	<0.01
ALT, median (IQR), U/L	89.8 (52.0-162.9)	93.8 (44.5-148.0)	0.735
AST, median (IQR), U/L	178.7 (97.5-338.5)	304.0 (85.9-554.3)	0.102
ALP, median (IQR), U/L	54.0 (48.4-70.9)	65.0 (51.9-90.6)	0.109
GGT, median (IQR), U/L	40.3 (26.4-69.3)	47.0 (26.4-87.9)	0.589
WBC, median (IQR), ×10^9^/L	2.1 (1.4-3.1)	1.7 (1.2-2.6)	0.253
Viral load, median (IQR), Log_10_ Copies/mL	4.6 (4.4-4.8)	4.5 (4.3-4.7)	0.178
PLT-Day3^b^, No. (%)			
Yes	52 (47.7)	2 (5.7)	<0.001
No	57 (52.3)	33 (94.3)	
PLT-Day5^c^, No. (%)			
Yes	65 (59.6)	2 (5.7)	<0.001
No	44 (40.4)	33 (94.3)	

^*^Except for the platelet, the detection time points of other biochemical indicators were at the at the time of admission.

^†^The first instance of the patient's platelet count dropping below 50×10^9^/L following admission is defined as Day1 PLT.

a APTT, activated partial thromboplastin time; PT, prothrombin time; ALT, alanine aminotransferase; AST, aspartate aminotransferase; CK, creatine kinase;

LDH, lactate dehydrogenase; ALP, alkaline phosphatase; GGT, γ-glutamyl transferase; WBC, white blood cell;

b whether the platelet count recovers to 40×10^9^/L within 3 days.

c whether the platelet count recovers to 50×10^9^/L within 5 days.

### Comparison of mortality risk between patients with recovered and unrecovered PLT on day 5

The Kaplan-Meier analysis indicated that patients with unrecovered PLT on day 5 had higher cumulative incidence of mortality than patients with recovered PLT on day 5 (**Figure 1**).

### Multicollinearity test

For the independent variables that show significant differences in univariate analysis (*p* < 0.05) and those deemed meaningful based on clinical experience, a multiple collinearity analysis was conducted. The results are shown in **Table 3**. The severity of collinearity between variables was measured using the Variance Inflation Factor (VIF), with VIF > 10 indicating significant collinearity between variables.

**Table 3:** Multicollinearity test.

Variable	Tol	VIF
Age ≥65 years	0.944	1.059
APTT	0.792	1.262
LDH	0.969	1.032
PLT Day3^a^	0.790	1.265
PLT Day5^b^	0.714	1.400
Whether platelets are transfused	0.862	1.160
Viral load (Log_10_ Copies/mL)	0.948	1.055

a whether the platelet count recovers to 40×10^9^/L within 3 days.

b whether the platelet count recovers to 50×10^9^/L within 5 days.

### Multivariate Cox regression analysis

To determine whether the selected variables are independent risk factors for mortality in severe SFTS patients, this study further constructed a multivariate Cox regression model. Combining the results of univariate analysis, collinearity tests, and clinical experience, relevant variables were included in the multivariate Cox regression analysis model to identify independent risk factors for mortality in severe SFTS. As shown in **Table 4**, LDH, APTT, age, and PLT-Day5 are independent predictors of mortality in severe SFTS patients. PLT-Day5 exhibited strong predictive power. Specifically, recovered PLT-Day5 can reduce mortality by 93% (*p* < 0.01).

**Table 4:** Multivariate cox regression analysis of risk factors for mortality in severe SFTS patients.

Variable	B	SE	Exp (B) (95% CI)	p
Age ≥65 years	0.772	0.390	2.164 (1.007 ~ 4.648)	0.048
APTT (s)	0.018	0.006	1.018 (1.006 ~ 1.030)	0.003
LDH (U/mL)	0.172	0.067	1.187 (1.040 ~ 1.355)	0.011
PLT-Day5 (×10^9^/L) ^a^	-2.604	0.762	0.074 (0.017 ~ 0.329)	<0.001

a whether the platelet count recovers to 50×10^9^/L within 5 days.

### Construct and validate a nomogram to predict the survival rate of severe SFTS patients

Based on the aforementioned risk factors, a nomogram was established to predict the mortality rates of patients with severe fever with thrombocytopenia syndrome (SFTS) (**Figure 2A**). The area under the curve (AUC) of this nomogram for predicting the 3-month mortality rate of severe SFTS patients is 88.1%. It is higher than the prediction models constructed by using PLT-Day5 alone (AUC = 75.6%, *p* = 7.23×10^−7^, DeLong’s test) or PLT-Day3 alone (AUC = 70.3%, *p* = 1.34×10^−5^, DeLong’s test) (**Figure 2B**). Further calibration curve analysis revealed a good consistency between the actual observed results and the nomogram predictions for the 3-month mortality probabilities (**Figure 2C**). The decision curve analysis (DCA) for the model is shown in **Figure 2D**. In this study, the DCA indicated that the model generated net benefits within a certain range of threshold probabilities.

### Subgroup analysis

The results of subgroup analyses are listed in **Figure 3**. A significant association between PLT-Day5 and mortality was observed in all subgroups. In addition, no significant interaction was observed between unrecovered PLT-Day5 and recovered PLT-Day5 groups in all strata.

## Discussion

SFTS, an emerging infectious disease, is predominantly endemic in China, Japan, and South Korea (Kim et al., 2013; Takahashi et al., 2014; Fang et al., 2015). From 2013 to October 2016, over 7,419 SFTS cases were reported in China (Zhan et al., 2017), with higher case fatality rates (CFRs) observed in Japan and South Korea than in China (He et al., 2020). Since 2019, SFTS cases have also emerged in Southeast Asia (Tran et al., 2019; Win et al., 2020; Zohaib et al., 2020; Rattanakomol et al., 2022), indicating the expanding geographical distribution of the SFTSV.

Current SFTS treatment primarily relies on antiviral and supportive therapies. Ribavirin, a potential anti-SFTSV agent, has been shown in retrospective studies to be ineffective in improving disease outcomes (Liu et al., 2013) or only marginally effective when administered early to patients with extremely low viremia (Li et al., 2018). Favipiravir (T-705), initially approved in Japan as an anti-influenza drug (Shiraki and Daikoku, 2020), has demonstrated therapeutic benefits for SFTS in a single-blind randomized controlled trial (Li et al., 2021); however, it is only effective in specific patient subgroups (aged ≤ 70 years, onset-to-admission interval ≤ 5 days, treatment duration ≥ 5 days, or baseline viral load ≤ 1×10^6^ copies/mL), and cautious use is recommended in patients over 70 years due to the lack of therapeutic benefit (Yuan et al., 2021). Mild SFTS cases achieve favorable prognoses with symptomatic treatment alone, while severe cases require targeted management of complications. Although high-dose gamma globulin administration (for critically ill patients with severe thrombocytopenia) and platelet transfusion (when necessary) have been proposed (Wei et al., 2022), our study found no significant difference in platelet transfusion rates between non-survivors and survivors (p > 0.05). Additionally, severely ill patients showed only slight or no improvement in platelet counts post-transfusion, consistent with findings by Li et al (Li et al., 2020), suggesting that platelet transfusion does not improve the prognosis of severe SFTS.

Thrombocytopenia is a core clinical feature of SFTS, and its severity predicts prognosis, for instance, a platelet counts < 50×10_9_/L is a risk factor for mortality (Cui et al., 2012). Previous studies have also identified older age, early-stage laboratory markers (lactate dehydrogenase, blood urea nitrogen, neutrophil percentage, aspartate aminotransferase), and neurological symptoms as prognostic indicators (Li et al., 2018). However, the role of platelet count recovery during the disease course remained unclear. Using a predictive model, our study is the first to demonstrate that, among severe SFTS patients with platelet counts dropping below 50×10^9^/L, those whose platelets recover from the nadir to ≥ 50×10^9^/L within 5 days of admission have better prognoses. Mechanistically, SFTSV induces hemophagocytosis in macrophages (Nakano et al., 2017), triggers cytokine storms (Jung et al., 2019), and replicates within platelets to facilitate dissemination (Fang et al., 2023)-contributing to persistent viremia and impaired platelet recovery. Conversely, platelets act as “sentinels” by rapidly endocytosing SFTSV particles to aid viral clearance, with a significant correlation observed between platelet levels and viral load (platelet counts reach their lowest level 3 days after peak viral load) (Yang et al., 2016; Fang et al., 2023). Delayed platelet recovery thus reflects pathological imbalances (inadequate viral clearance, excessive immune activation, and SFTSV utilization of platelets for replication), explaining its association with poor prognosis.

Compared with viral load, platelet count recovery offers distinct advantages as a monitoring indicator. Viral load detection is time-consuming, limited to select local centers for disease control, and undetectable in 55.6% of confirmed patients at admission (Yang et al., 2016). In contrast, platelet counts can be measured conveniently and rapidly, making them more clinically applicable.

This study has several limitations. First, its retrospective design may have led to incomplete data recording and recall bias. Second, the small sample size (limited to severe SFTS cases) restricts statistical power and generalizability, with conclusions applicable only to severely ill hospitalized patients rather than the broader SFTS population. Third, the absence of dynamic viral monitoring data prevented direct verification of the “quantitative association between platelet recovery rate and viral clearance efficiency,” obscuring the precise pathway linking “platelet dynamics-viral kinetics-prognosis.” Future studies should (1) incorporate longitudinal viral load monitoring to clarify the relationship between platelet recovery rate and viral clearance, and (2) explore the molecular mechanisms underlying platelet-mediated viral clearance to validate why platelet recovery serves as an independent prognostic indicator.

In conclusion, recovery of platelet count is an independent prognostic factor for severe SFTS patients with platelet count lower than 50×10^9^/L. Those who showed platelet recovery had lower mortality than those who did not. Whether platelet recovery plays the same role in all severe SFTS patient needs further study in a sufficiently large number of SFTS patients.

## Data Availability

The original contributions presented in the study are included in the article/Supplementary Material. Further inquiries can be directed to the corresponding authors.
